# Effects of Fluoride on Two Chemical Models of Enamel Demineralization

**DOI:** 10.3390/ma10111245

**Published:** 2017-10-27

**Authors:** Ollie Yiru Yu, May Lei Mei, Irene Shuping Zhao, Edward Chin-Man Lo, Chun-Hung Chu

**Affiliations:** Faculty of Dentistry, The University of Hong Kong, Hong Kong, China; yuyiru@hku.hk (O.Y.Y.); mei1123@hku.hk (M.L.M.); irenezhao110@gmail.com (I.S.Z.); hrdplcm@hkucc.hku.hk (E.C.-M.L.)

**Keywords:** demineralization, enamel, fluoride

## Abstract

This study evaluated the effects of fluoride on subsurface enamel demineralization induced by two commonly used chemical models. Forty-eight enamel blocks were demineralized at pH = 5.0 by an acetate buffer (Group 1), a lactate buffer (Group 2), an acetate buffer with 0.02 ppm fluoride (Group 3) and a lactate buffer with 0.02 ppm fluoride (Group 4) at 25 °C for 3 weeks. The surface destruction percentage (SDP), mineral loss and lesion depth of the blocks were studied using micro-computed tomography. An elemental analysis of the enamel surface was evaluated using an energy-dispersive X-ray spectroscopy. Surface micro-hardness was determined by the Knoop Hardness Test. The mean lesion depth of Groups 1 through 4 were 134.1 ± 27.2 μm, 96.1 ± 16.5 μm, 97.5 ± 22.4 μm and 91.1 ± 16.2 μm, respectively (*p* < 0.001; group 1 > 2, 3 > 4). The SDPs of groups 1 through 4 were 7.8 ± 8.93%, 0.71 ± 1.6%, 0.36 ± 1.70% and 1.36 ± 2.94% (*p* < 0.001; group 1 > 2, 3, 4). The fluoride in mean weight percentages of groups 1 through 4 were 1.12 ± 0.24%, 1.10 ± 0.20%, 1.45 ± 0.40% and 1.51 ± 0.51%, respectively (*p* < 0.001; group 3, 4 > 1, 2). The mean Knoop hardness values of groups 1 through 4 were 27.5 ± 13.3, 39.7 ± 19.3, 73.6 ± 44.2 and 91.0 ± 57.2, respectively (*p* < 0.001; group 4 > 3 > 2 > 1). The chemical model using an acetate buffer solution created significantly deeper zones of subsurface demineralization on enamel than the lactate buffer solution. An acetate buffer may damage the enamel surface, but the surface damage can be prevented by adding fluoride.

## 1. Introduction

Although enamel is the most acid-resistant substance in the human body, it is constantly subjected to the presence of cariogenic plaque along with the presence of fermentable carbohydrates. The demineralization process happens when the environmental acidity (pH) drops below a certain level (critical pH) [1]. The main component of enamel is the hydroxyapatite crystal composition of the enamel prism. The columnar prisms are the basic structures of enamel. The space between the columnar prisms is filled with organic components and water. Saliva and plaque fluid are not saturated with calcium and phosphate when the pH drops. This is when the dissolution of the enamel happens. As the demineralization goes on, the substantial deficiency appears. The construction of the enamel is different from dentine. Mineral components make up 85% of enamel’s volume, while the other 15% consists of organic components and water [2]. This is different from the structure of dentine, in which the matrix is comprised of collagen. The inter-prism substance of enamel is not strong enough to sustain the framework. Therefore, it is easier to find substantial deficiencies in enamel demineralization models. In current cariology research, one important way to observe the effect of remineralization is to evaluate the density of demineralized enamel tissue. However, once the substantial deficiency occurs, the effect of arresting caries and enamel tissue remineralization are difficult to be observed. Thus, when creating carious lesions, it is important to create subsurface lesions, which are lesions with intact surface.

A recent review found in vitro studies were the most common mechanistic studies on cariology [3]. Among these in vitro studies, most studies used simple mineralization chemical models to generate artificial carious lesions [3]. These models employ acidic demineralization agents to generate demineralized lesions. Mild organic acids and acid buffers such as lactic acid and acetate acid are the most commonly used acid to create demineralized lesions. These acid buffers can create demineralized lesions to mimic caries lesions. Generally, a solution with a stable pH value is used to create artificial caries. The acidity to create a subsurface lesion ranges from pH 4.4 to 5.0 in most studies [3]. They have obvious advantages such as time and cost saving, controllable experimental conditions, reproducibility of the experiment and simplicity of the studies [4]. In in vitro chemical models, the demineralization process is simplified to the interaction between substrates and acid—the metabolic production of biofilm. The properties of the caries-like lesions can be regulated by factors such as pH, time, temperature, mineral concentration and presence of mineral dissolution inhibitors [5]. By modifying these factors, the characteristics of lesions such as lesion depth, mineral loss ratio and distribution of mineral lost can be controlled [6]. A chemical model is a compromise between the reality of the in vivo ecosystem and the simplification of the system. Recent studies have compared carious lesions created by in vitro chemical protocol to natural carious lesions. The result show that artificial caries induced by chemical models exhibited several characteristics similar to natural caries [6]. Hence, these lesions were regarded as acceptable and were used in a lot of cariology research to create enamel lesions.

Fluoride is commonly used for caries control. The presence of fluoride in saliva makes it a natural remineralization solution. Even a low concentration of fluoride is effective in interrupting the demineralization process. When the pH drops below 5.5 but remains higher than 4.5, the hydroxyapatite dissolves and fluorapatite starts to generate [7]. As the solubility of the fluorapatite is lower than that of hydroxyapatite, the enamel dissolution process will slow down. The enamel’s continuously lost calcium and phosphorus will be recovered as fluorapatite [8]. Hence, to decrease the severity of the destruction to the demineralized tissue, some researchers add fluoride into the demineralization solutions to create subsurface lesions [9,10]. However, the effect of fluoride on different chemical caries models has not been previously explored. The purpose of the study is to evaluate the effect of low concentrations of fluoride on two commonly used chemical models.

## 2. Results

**Figure 1:**
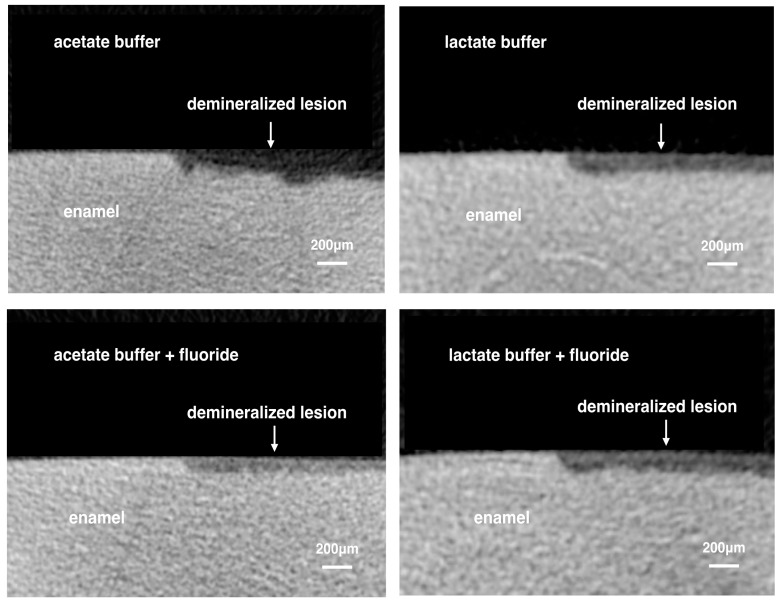
Representative images of reconstructed micro-computed tomography of the four experimental groups.

Table 1 shows the mean lesion depth, mineral loss and surface destruction percentages of the four experimental groups. The reconstructed micro-computed tomography (micro-CT) images of the four groups are present in Figure 1.

**Figure 2:**
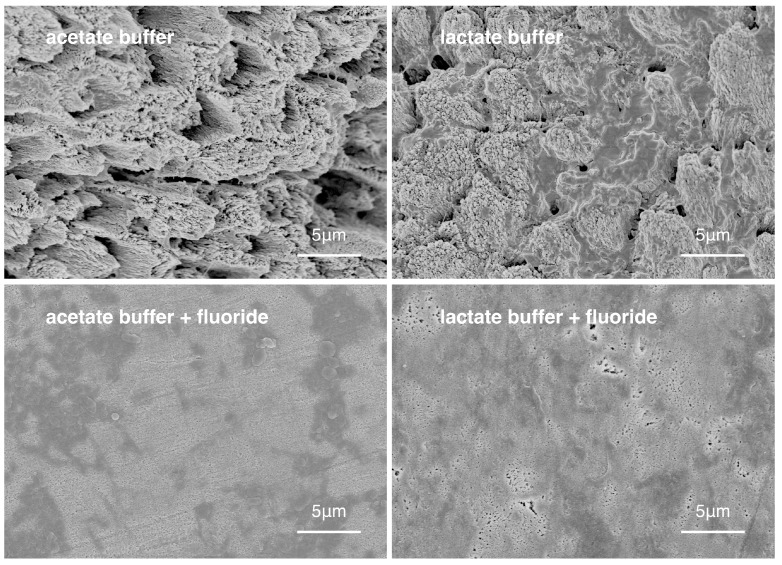
Surface morphology of enamel of the four experimental groups.

Representative scanning electronic microscope (SEM) images showing the enamel surface morphology of the four groups are shown in Figure 2. SEM/energy-dispersive X-ray spectroscopy (EDX) showed that the fluoride in the mean weight percentage (±SD) of groups 1 through 4 were 1.12 ± 0.24%, 1.10 ± 0.20%, 1.45 ± 0.40% and 1.51 ± 0.51%, respectively (*p* < 0.001; group 3, 4 > 1, 2). The mean Knoop hardness values (±SD) of group 1 to 4 were 27.5 ± 13.3, 39.7 ± 19.3, 73.6 ± 44.2, 91.0 ± 57.2, respectively (*p* < 0.001; group 4 > 3 > 2 > 1).

## 3. Discussion

Chemical models simplify the caries formation process to a pure demineralization process, because they use simple demineralization agents of low pH value (usually acid) to demineralize enamel [3]. Acid buffers are commonly used to create artificial caries lesions because they can create demineralized enamel lesions that are more similar to natural caries than inorganic acids. In general, one single solution with a stable pH value is used to create artificial caries. This method was used by many researchers, because it saves time and because the experiment operation is straightforward. Another advantage of this method is that the extent of demineralization can be controlled by adjusting the conditions including acidity, temperature and duration of the demineralization [5]. The pH value of the demineralization solutions used mostly ranged from 4.4 to 5.0, according to the study designs. A pH at 5.0 was chosen in this study to prevent the unwarranted demineralization of the enamel surface. Like most chemical models, this model could induce a higher mineral loss ratio than natural caries [6]. Furthermore, the basic design of this chemical model is simple and cannot simulate the complicated process of natural caries development.

Lactic acid and acetate acid are the common acid-buffer solutions used in chemical models to create demineralized lesions or artificial caries for cariology research [11,12,13]. Hence, they were chosen in this study. It was suggested that lactate acid could dominate in active caries, while acetate acid was often associated with arrested caries lesions. An in vitro study showed that lactic acid was more effective than other organic acids for demineralization and creating carious lesion [14]. Lactic acid with an acid dissociation constant (pKa) of 3.86 is lower than that of acetic acid (pKa = 4.76) at 25 °C. However, this study found that the acetate buffer created deeper lesions than the lactate buffer at the same pH value. This might be because the unionized acid concentration of acetic acid is higher than that of lactic acid [14]. The unionized acid could diffuse and get into the enamel. Then, it could continuously release hydrogen ions (H^+^) and dissolve the hydroxyapatite crystal. Another reason might be the use of tetraethyl methyl diphosphonate. It is a dissolution inhibitor that prevents the aggressive demineralization of enamel by lactate acid [15].

The physical and mechanical properties of these artificial carious lesions would influence the subsequent demineralization and remineralization process and even the results of the experiment [16]. In clinical situations, an early carious lesion on the smooth surface of enamel is manifested as a white spot lesion. Histologically, they might be classified into four zones: surface layer, body of lesion, dark zone and translucent zone [17]. The surface layer formation in natural caries lesions might be caused by the presence of demineralization inhibitors such as the fluoride and proteins in saliva [18]. The fluoridation of drinking water and the use of fluoridated anti-caries products such as fluoride toothpaste are the two common sources of fluoride. The fluoride is stored in the oral cavity and released slowly [19]. Fluoride can also be found in saliva, but the concentration is very low and normally at a sub-ppm level. It has been suggested that 0.02 ppm fluoride was involved in surface layer formation of enamel lesion in in vivo conditions [20]. In this study, the enamel blocks were continuously subject to acid challenge. Because of the absence of the remineralization process or a demineralization inhibitor, the subsurface porosity kept developing and resulted in the consolidation of porosities and cavitation [21]. Susceptible enamel became increasingly vulnerable in this process. Finally, the carious enamel tissue collapsed and exposed the irregular surface. However, in the presence of low concentrations of fluoride, the carious formation process slowed down. The demineralization process continued, but remineralization took place on the enamel block’s surface. As a result, a surface layer with a higher mineral density than the lesion body was formed. Thus, adding low concentrations of fluoride to a demineralizing solution of a chemical caries model could generate a caries-like lesion, which simulates natural carious lesions. EDX and Knoop micro-hardness testing were used to compare the surface differences of the specimens in the study’s four experimental groups. The results confirmed that the fluoride content on the enamel surface of the demineralized lesion increased when fluoride was added to the acid buffer solutions.

In this study, the addition of fluoride to the acetate buffer solution had a more profound demineralizing effect than that in the lactate buffer. This may have contributed to the higher dissolution rate of acetic acid. While the hydroxyapatite dissolved, the fluorapatite might have formed in the remineralization-demineralization process. In addition, the lactate buffer solution contained tetraethyl methyl diphosphonate (TEMPD), which is an enamel demineralization inhibitor and could have slowed down the dissolution rate of hydroxyapatite.

The results of the lesion depth and surface loss percentages showed that, among the four experimental groups, the acetate buffer solution created the deepest lesions. However, it also created significantly greater surface loss than the other three experimental groups. The results concurred with previous studies, which found that the properties of caries-like lesions could be controlled [6,16,22]. This study found that fluoride could prevent damage to the enamel surface by the acid challenge. The addition of fluoride and TEMDP would affect the presence of the surface layer and the rate of dissolution. Thus, the extent of demineralization could be manipulated [6,16,22].

The physical and mechanical properties of the artificial lesions might be different if the concentration of the fluoride changed [23]. Other conditions such as the use of deciduous or permanent teeth might also affect the result. Studies suggested that the micro-hardness, shear bond strength and shear resistance of the specimen were different between deciduous and permanent teeth [24,25,26]. The effect of erosion and acidic primer were also dissimilar on these two kinds of teeth [27,28]. Thus, the demineralization of the specimen might be altered if deciduous teeth were chosen in the present study. The results of the present report are promising but further studies testing different variables are needed to confirm the role of fluoride in demineralization experiments.

## 4. Materials and Methods

### 4.1. Preparation of Enamel Blocks

This study was approved by the Institutional Review Board of the University of Hong Kong (IRB UW133-22). Extracted human sound third molars were collected with the patient’s consent.

The molars were stored in a 0.5% thymol solution at 4 °C before its use (pH 7.0). Enamel slices with 2 mm thickness were prepared from the molars. Using sanding paper of micro-fine 4000 grit, the enamel surfaces of the slices were polished until smooth. Each slice was sectioned into four enamel blocks, which were allocated into four treatment groups for the experiment. Each set of the four enamel blocks were individually examined with a stereomicroscope (×10). The set of four enamel blocks were excluded if cracks or other defects such as enamel opacity or hypoplasia were found. The proper enamel blocks were half covered with acid-resistant nail polish (Clarins, Paris, France) to create a self-control region as a reference for the evaluation.

The four enamel blocks prepared from the same enamel slice were allocated into four treatment groups for demineralization. Blocks allocated into Group 1 were demineralized with an acetate buffer solution (pH 5.0) containing 50 mM acetate buffer, 2.2 mM calcium chloride (CaCl_2_), 2.2 mM potassium dihydrogen phosphate (KH_2_PO_4_) and 0.02% sodium azide (NaN_3_) [29]. Blocks in Group 2 were demineralized with a lactate buffer solution (pH 5.0) containing 50 mM lactate buffer, 3 mM CaCl_2_, 3 mM KH_2_PO_4_, 6 μM TEMDP and 0.02% NaN_3_ [15]. Blocks in Group 3 were demineralized with the acetate buffer solution (Group 1) with sodium fluoride (NaF) with a fluoride (F) concentration at 0.02 ppm. The blocks in Group 4 were demineralized with the lactate buffer solution with NaF with 0.02 ppm F. Each enamel block was immersed in 1 mL of respective solution in individual well at 25 °C for 21 days. The demineralizing solution was refreshed regularly every 24 h. Twelve enamel blocks were demineralized in each experimental group for assessment.

### 4.2. Assessment of Enamel Demineralization

#### 4.2.1. Lesion Depth, Mineral Loss and Surface Destruction

Lesion depth, mineral loss and surface integrity of the enamel blocks were evaluated by X-ray micro-computed tomography (Micro-CT) (SkyScan 1172; SkyScan, Antwerp, Belgium). The X-ray source was operated at a source voltage of 80 kV and a current of 100 μA. The pixel size of the image was set as 6 μm. A 0.5 mm aluminium filter was used to cut off the softest X-rays. The scanning results for each specimen were reconstructed using the NRecon reconstruction software (SkyScan, Antwerp, Belgium). The reconstructed three-dimensional images were viewed and processed using the data analysing software, CTAn (SkyScan, Antwerp, Belgium). Approximately 600 cross-sectional images of each enamel block were obtained from the reconstructed three-dimensional image. Twenty images were randomly selected from these cross-sectional images for assessment. An image area with a grayscale value of more than 95% of the untreated enamel (internal control) was defined as sound enamel [30]. Software (Image J; National Institutes of Health, MD, USA) with a plot profile was used for the image analysis. The areas of demineralized enamel were determined, and the depth of the lesions was measured.

**Figure 3:**
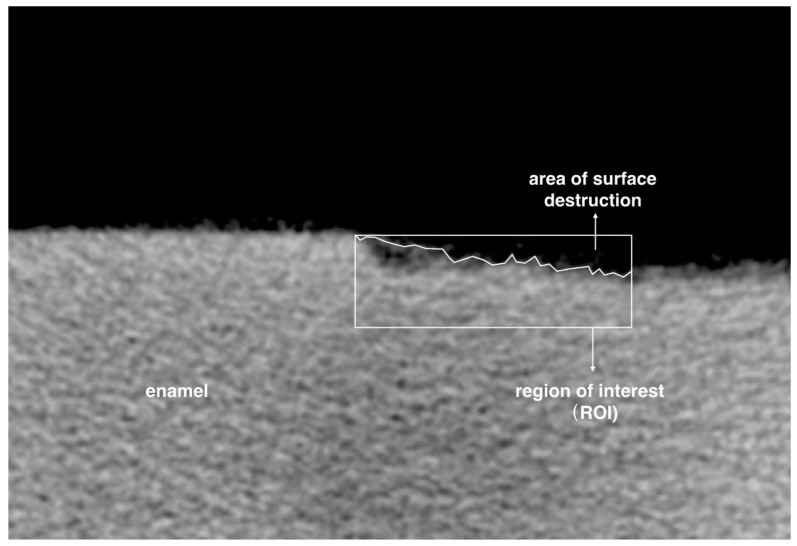
Illustration of choosing surface destruction area. Surface destruction percentage = area of surface destruction/area of region of interest.

The greyscale value was calculated into mineral density value (MDV, g_HAp_·cm**^−^**^3^) by CTAn. Two mineral cylindrical phantoms with the MDVs of 0.25 g_HAp_·cm**^−^**^3^ and 0.75 g_HAp_·cm**^−^**^3^ were used for the calibration. The mean MDV of the demineralized area of each specimen was evaluated. Mineral loss (ΔZ; g_HAp_·cm**^−^**^3^) was calculated by subtracting the MDV of the demineralized area from the MDV obtained from the area of sound enamel before demineralization [31]. A region of interest (ROI) of the Micro-CT image with an area of 720 μm in width and 240 μm in depth was chosen to assess the enamel surface destruction (Figure 3). Five ROIs were selected from each enamel block. The greyscale value of more than 40% was set as remaining enamel tissue. The surface destruction percentage (SDP), which was the ratio of the surface destruction area and ROI, was calculated by CTAn.

#### 4.2.2. Elemental Analysis

The enamel blocks were ultrasonically washed in distilled water three times, dehydrated in a series of ethanol solutions, dried in a desiccator and finally sputter-coated with carbon. The surface morphology of the enamel blocks was then examined under a scanning electron microscopy (SEM) (Hitachi S-4800 FEG Scanning Electron Microscope, Hitachi Ltd., Tokyo, Japan) at 5 kV in high-vacuum mode. An elemental analysis was then carried out to study the fluoride (F) ions on the enamel lesion surface by an energy-dispersive X-ray spectroscopy (EDX) under SEM. The elemental analysis was performed by measuring three areas (5 × 5 μm^2^) on the surface of each enamel block, and the mean weight percentages of F were calculated.

#### 4.2.3. Surface Micro-Hardness

Surface micro-hardness of the enamel blocks was tested by a Knoop Hardness Tester (Leitz, Micro-hardness Tester; Ernst Leitz Wetzlar GmbH, Wetzlar, Germany) after 3 weeks of demineralization. The enamel block was placed under the Knoop indenter of the tester. Twenty indentations were made on the intact area of the lesion surface side of each enamel block with a load of 5 gf (49 × 10^−3^ N) for 10 s at each test point [32]. The indentations were approximately 100 μm from each other. The mean Knoop hardness numbers (KHN) were calculated for reporting and analysis.

#### 4.2.4. Statistical Analysis

Our pilot study found that the mean lesion depth of the test group was approximately 100 μm. This study aimed to detect a difference of at least 20 μm. Assuming a common standard deviation of 20 μm and with power at 0.8, the sample size required at least ten enamel blocks in each group. Statistical analyses were conducted using IBM SPSS Statistics 22 software (IBM Corporation, Armonk, NY, USA). All data were assessed using the Shapiro-Wilk test for normality (*p* > 0.05). One-way ANOVA test was performed. Bonferroni post hoc test was applied to compare the lesion depth, mineral loss, surface destruction percentage and fluoride concentration among the 4 experimental groups. The significance level was set at 5%.

## 5. Conclusions

The effect of fluoride on demineralized enamel lesions created by a lactate buffer is not significant. The surface integrity of the demineralized enamel lesion created by an acetate buffer can be preserved in the presence of fluoride. Adding fluoride to chemical models using an acetate buffer is recommended when creating artificial carious lesions within the limitations of the present report.

## Figures and Tables

**Table 1:** Mean lesion depth, mineral loss and surface destruction percentage of the four experimental groups.

Enamel Demineralization	Acetate Buffer	Lactate Buffer	Acetate Buffer with Fluoride	Lactate Buffer with Fluoride	*p*-Value Bonferroni Test
Lesion depth (μm)	134.1 ± 27.2 ^a^	96.1 ± 16.5 ^b^	97.5 ± 22.4 ^b^	91.1 ± 16.2 ^c^	<0.001 a > b > c
Mineral loss (g·cm^−3^)	1.18 ± 0.21 ^a^	1.13 ± 0.27 ^a^	0.87 ± 0.29 ^b^	1.06 ± 0.22 ^a^	<0.001 a > b
Surface destruction (%)	7.8 ± 8.93 ^a^	0.71 ± 1.6 ^b^	0.36 ± 1.70 ^b^	1.36 ± 2.94 ^b^	<0.001 a > b

## Abbreviations

The following abbreviations are used in this manuscript:

SDP	surface destruction percentage
CaCl_2_	calcium chloride
KH_2_PO_4_	potassium dihydrogen phosphate
NaN_3_	sodium azide
TEMDP	tetraethyl methyl diphosphonate
NaF	sodium fluoride
F	fluoride
Micro-CT	micro-computed tomography
MDV	mineral density value
ROI	region of interest
SEM	scanning electron microscopy
EDS	energy-dispersive X-ray spectroscopy
KHN	Knoop hardness number
